# Detection of ^13^C labeling of glutamate and glutamine in human brain by proton magnetic resonance spectroscopy

**DOI:** 10.1038/s41598-022-12654-y

**Published:** 2022-05-24

**Authors:** Li An, Shizhe Li, Maria Ferraris Araneta, Christopher S. Johnson, Jun Shen

**Affiliations:** Molecular Imaging Branch, National Institute of Mental Health, National Institutes of Health, Building 10, Room 3D46, 10 Center Drive, MSC 1216, Bethesda, MD 20892-1216 USA

**Keywords:** Magnetic resonance imaging, Disorders of consciousness, Magnetic resonance imaging

## Abstract

A proton magnetic resonance spectroscopy (MRS) technique was used to measure ^13^C enrichments of glutamate and glutamine in a 3.5 × 1.8 × 2 cm^3^ voxel placed in the dorsal anterior cingulate cortex of five healthy participants after oral administration of [U-^13^C]glucose. Strong pseudo singlets of glutamate and glutamine were induced to enhance the signal strength of glutamate and glutamine. This study demonstrated that ^13^C labeling of glutamate and glutamine can be measured with the high sensitivity and spatial resolution of ^1^H MRS using a proton-only MRS technique with standard commercial hardware. Furthermore, it is feasible to measure ^13^C labeling of glutamate and glutamine in limbic structures, which play major roles in behavioral and emotional responses and whose abnormalities are involved in many neuropsychiatric disorders.

## Introduction

Noninvasive in vivo detection of ^13^C labeling of glutamate (Glu) and glutamine (Gln) is a powerful tool for investigating Glu and Gln metabolism and neurotransmission in the brain^1^. Two types of ^13^C magnetic resonance spectroscopy (MRS) techniques have been widely used: direct ^13^C MRS and indirect ^1^H-[^13^C] MRS. Due to the much lower sensitivity of ^13^C nuclei, direct ^13^C MRS generally requires surface coils and very large tissue volume to achieve adequate signal-to-noise ratio (SNR). Hence, only the neocortex is accessible to direct ^13^C MRS experiments. The indirect ^1^H-[^13^C] MRS techniques make use of the ^1^H-^13^C coupling and difference spectroscopy to detect signals from protons bound to ^13^C^2–9^. Because of the larger gyromagnetic ratio of ^1^H compared to ^13^C, these indirect ^13^C detection techniques have higher sensitivity compared to the direct ^13^C detection techniques. Broadband magnetic resonance imaging (MRI) scanners equipped with heteronuclear capabilities are required to perform either direct ^13^C or indirect ^1^H-[^13^C] MRS. To enhance sensitivity and make heteronuclear nuclear Overhauser enhancement/decoupling feasible, the RF coil assembly needs to be high-efficiency non-volume ^13^C and ^1^H coils, which is a non-standard device and not commercially available. Because of these technical barriers, ^13^C MRS of human brain has been largely confined to only a few research groups with very limited clinical applications so far.

Attempts have been made to circumvent the hardware limitation of ^13^C MRS by measuring signal changes in short echo time (TE) ^1^H MRS spectra caused by incorporation of ^13^C labels into brain amino acids^10^. However, it has been difficult to reliably separate Glu and Gln in the crowded ^1^H MRS spectra. With the incorporation of ^13^C labels, the short TE ^1^H MRS spectra become even more complex. Due to these obstacles, ^13^C labeling of both Glu and Gln, the most important components of the glutamatergic system detectable by MRS, has been difficult to measure in the human brain using proton-only MRS methods.

Previously, our laboratory developed an MRS sequence for measuring Glu and Gln at 7 Tesla with minimized N-acetyl-aspartate (NAA) multiplet at 2.48 ppm using a J-suppression pulse^11^. This sequence was used to study the dynamic ^13^C labeling of Glu and Gln in the prefrontal cortex of healthy participants after intravenous infusion of [U-^13^C_6_]glucose^11,12^. However, the signal intensity of Gln at TE = 106 ms was low, hampering accurate detection of its ^13^C labeling.

Recently, our laboratory developed a 7 Tesla pulse sequence which can reliably measure Gln at TE = 56 ms with significantly improved sensitivity^13,14^. In this work, we demonstrate the feasibility of measuring ^13^C labeling of Glu and Gln using ^1^H MRS technique (TE = 56 ms) with a commercial proton-only head coil at 7 Tesla. Since this is a proton-only technique, no broadband capability or any custom-made hardware is necessary. In addition, because of the much higher sensitivity of proton MRS, we will also demonstrate for the first time, detection of ^13^C labeling of both Glu and Gln from an area in the limbic system. Specifically, using oral administration of ^13^C-labeled glucose, we will show that ^13^C labeling of Glu and Gln can be measured with high precision from the dorsal anterior cingulate cortex (dACC), a limbic region involved in cognition and motor control but is beyond the reach of conventional ^13^C MRS that relies on surface coils. It is hoped that the demonstration of measuring ^13^C-labeling of Glu and Gln with the high sensitivity and spatial resolution of proton MRS using commercial scanners and RF coils will generate interest in further improving MRS technology and greatly facilitate the adoption of ^13^C MRS strategies for probing energy metabolism and glutamatergic neurotransmission in clinical research.

## Results

**Figure 1:**
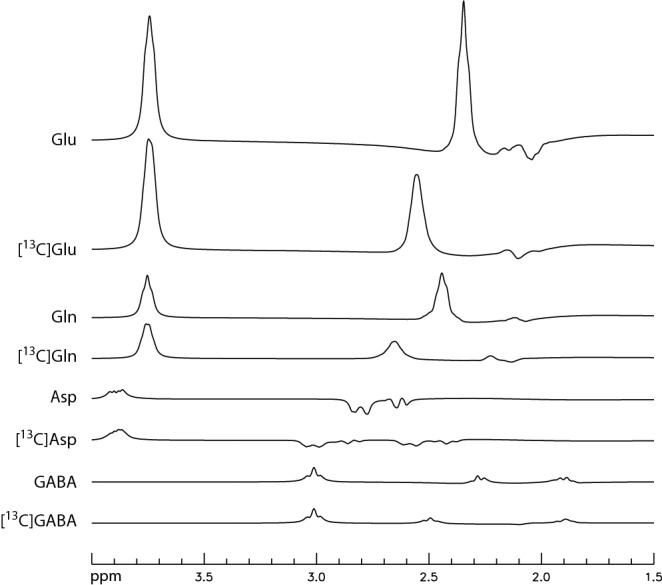
Numerically simulated spectra of Glu, ^13^C satellites of Glu ([^13^C]Glu), Gln, ^13^C satellites of Gln ([^13^C]Gln), Asp, ^13^C satellites of Asp ([^13^C]Asp), GABA, and ^13^C satellites of GABA ([^13^C]GABA) at TE = 56 ms. The corresponding concentration ratios were set to 10:10:3:3:3:3:1:1. The spectra were broadened to a singlet width of 8 Hz.

Figure 1 shows the calculated spectra of Glu, ^13^C satellites of Glu ([^13^C]Glu), Gln, ^13^C satellites of Gln ([^13^C]Gln), Asp, ^13^C satellites of Asp ([^13^C]Asp), GABA, and ^13^C satellites of GABA ([^13^C]GABA). As shown in Fig. 1, the spectra of Glu and Gln at TE = 56 ms are dominated by their respective H4 and H2 pseudo singlets. The ^13^C satellite signals of Glu H4 in the proton channel, [^13^C]Glu, are resulted from the large one-bond scalar coupling between the H4 pseudo singlet and the ^13^C label at C4. As both carbons of the acetyl CoA are ^13^C-labeled after administration of uniformly labeled glucose^15^, [4,5-^13^C]Glu was used as the starting point for spectral fitting of Glu ^13^C satellites in this study. Similarly, [4,5-^13^C]Gln and [3,4-^13^C]Asp were chosen as the starting points for fitting the ^13^C satellites of Gln H4 and Asp H3, respectively. The [4,5-^13^C]Glu, [4,5-^13^C]Gln and [3,4-^13^C]Asp spectra in Fig. 1 were generated using one-bound and long-range ^1^H-^13^C coupling constants reported in the literature^16^ except for the long-range ^1^H-^13^C coupling constants involving the carboxylic carbons which, to the best of our knowledge, were not available. Instead, the corresponding long-range ^1^H-^13^C coupling constants involving C4 (C3 for Asp) were used as their substitutes. The actual values of the long-range ^1^H-^13^C couplings used in the spectral model are not important as the spectral fitting program fits the in vivo spectra by adjusting the lineshape and linewidth of the ^13^C satellites to account for changes in B_0_ inhomogeneity and additional ^1^H-^13^C scalar couplings. Furthermore, although ^31^C label is transferred from glutamate C4 to GABA C2 during GABA formation, [2-^13^C]GABA was omitted in spectral fitting because the spectra of [2-^13^C]GABA and GABA are very weak compared to the resonance signals of Glu H4, Gln H4, and their ^13^C satellites, as shown in Fig. 1. Note that the ^13^C satellite spectra of Glu H4 and Gln H4 in Fig. 1 are highly asymmetrical and dominated by a single downfield satellite peak.

**Figure 2:**
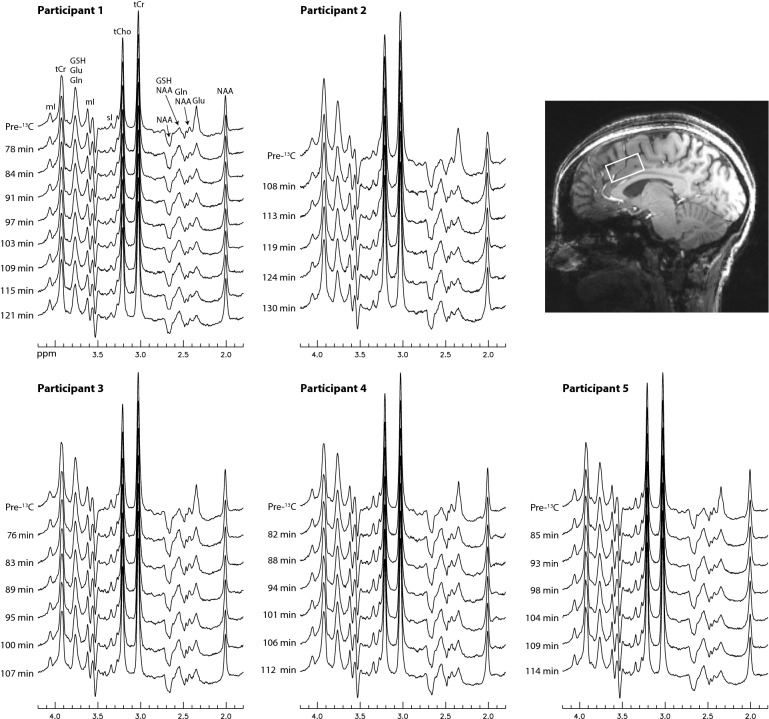
Time-course ^1^H spectra from the dorsal anterior cingulate cortex (dACC) of five healthy participants. No linebroadening was applied to the in vivo spectra. The listed times are the mid-point times of the MRS scans. Voxel size = 3.5 × 1.8 × 2 cm^3^; TR = 2.2 s; TE = 56 ms; spectral width = 4000 Hz; number of data points = 1024; number of averages = 264 and total scan time = 10 min for the pre-^13^C spectra; number of averages = 132 and total scan time = 5 min for each individual post-^13^C spectrum. The time course acquisition was initiated after the participants drank the glucose solution, rested, and then re-entered the magnet.

**Figure 3:**
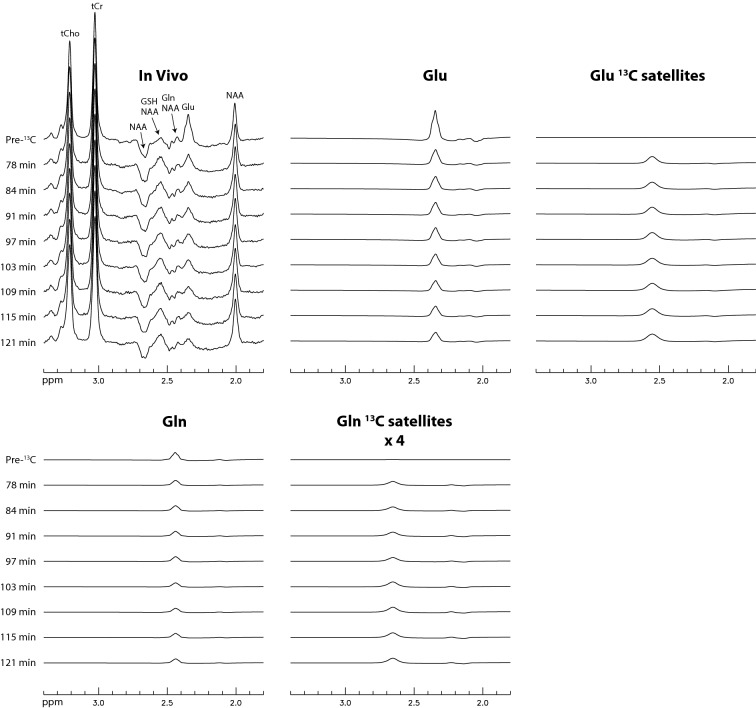
Time-course spectra and corresponding fitted spectra of Glu, Gln, and their ^13^C satellites acquired from the dACC of a representative participant. The spectra for Gln ^13^C satellites have been scaled up vertically by a factor of 4.

**Figure 4:**
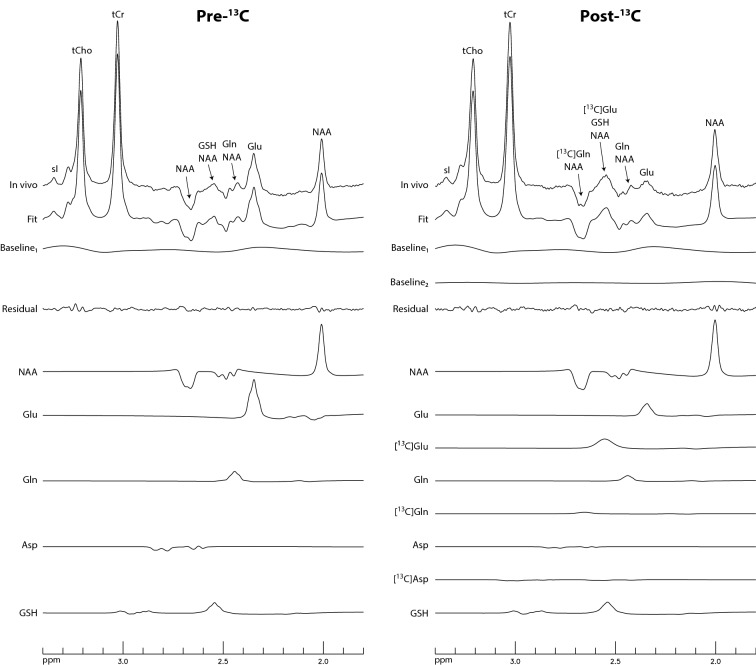
In vivo spectra and corresponding fitted spectra for the pre-^13^C MRS scan and the last post-^13^C scan of the participant in Fig. 3. Baseline_1_ is the baseline in the pre-^13^C spectrum, which is a spline baseline with 13 knots. The baseline in the post-^13^C spectrum is baseline_1_ + baseline_2_, in which baseline_2_ is a much weaker spline baseline with 8 knots.

Time-course ^1^H spectra from the dACC of the five participants are displayed in Fig. 2. The spectra are highly consistent with the spectral patterns by the numerical simulations. The Glu H4 peak at 2.34 ppm dropped dramatically after oral administration of [U-^13^C]glucose and, correspondingly, the peak at 2.56 ppm significantly increased due to the rise of the downfield ^13^C satellite signals of Glu H4. Figure 3 displays the time-course spectra of a representative participant and corresponding fitted spectra of Glu, Gln, and their ^13^C satellites. In the fitted Gln time-course spectra, the drop in peak amplitude of Gln H4 after oral administration of [U-^13^C]glucose can be clearly seen. Meanwhile, the rise of the peak for Gln ^13^C satellites (vertically scaled up by a factor of 4) after oral administration of [U-^13^C]glucose is apparent. The spectra and corresponding fits for the pre-^13^C MRS scan and the last post-^13^C scan of the participant are displayed in Fig. 4 with good fitting results. The spline baseline obtained by fitting the pre-^13^C spectrum was labeled as baseline_1_. To account for the participant repositioning (see “Methods” section), a second much weaker baseline (baseline_2_) was used, which was determined when fitting the post-^13^C spectrum. The total baseline for the post-^13^C spectrum was the sum of baseline_1_ and baseline_2._

**Figure 5:**
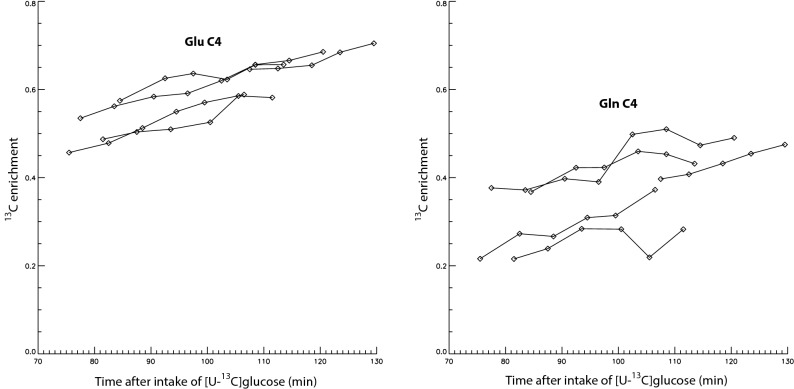
Plots of ^13^C enrichments of Glu C4 and Gln C4 vs. time after oral administration of [U-^13^C]glucose for all five healthy participants.

Figure 5 displays the plots of ^13^C enrichments of Glu C4 and Gln C4 vs. time after oral administration of [U-^13^C]glucose for all five participants. The behavior of the time course of ^13^C-labled Glu and Gln shown in Fig. 5 is consistent with them approaching maximum ^13^C labeling as expected from the time scale of cerebral Glu and Gln turnover^1^. Similar behavior was also observed in our previous direct ^13^C MRS experiments in the carboxylic/amide spectral region following oral administration of [U-^13^C]glucose^17^.

Metabolite ratios (/[tCr]) in the dACC of the five participants quantified from the pre-^13^C spectrum of each participant are given in Table 1. The results are highly consistent with our earlier ^1^H-only MRS study of the same brain region using the same pulse sequence^13^. Using the 12.6 mL (3.5 × 1.8 × 2 cm^3^) voxel size and 10 min scan time, the CRLB values for Glu and Gln were found to be 1.6 ± 0.2% for Glu and 3.2 ± 0.4% for Gln, indicating excellent precision. ^13^C enrichments of Glu C4 and Gln C4 for the five participants computed from the last two post-^13^C spectra of each participant, acquired at 113 ± 9 min after oral administration of [U-^13^C]glucose, were found to be 64 ± 5% for Glu and 40 ± 10% for Gln.

**Table 1:** Metabolite ratios (/[tCr]) in the dACC of five healthy participants quantified from the pre-^13^C spectrum of each participant.

	Metabolite ratio (/[tCr])	CRLB (%)
NAA	1.33 ± 0.09	1.2 ± 0.3
Glu	1.16 ± 0.08	1.6 ± 0.2
Gln	0.33 ± 0.07	3.2 ± 0.4
GSH	0.30 ± 0.02	3.0 ± 0.4
Asp	0.37 ± 0.07	8.0 ± 3.6
tCr	1	0.7 ± 0.2
tCho	0.25 ± 0.02	0.8 ± 0.2

Voxel size = 3.5 × 1.8 × 2 cm^3^; TR = 2.2 s; TE = 56 ms; spectral width = 4000 Hz; number of data points = 1024; number of averages = 264; and total scan time = 10 min.

Supplementary Figure S1 online shows the time-course difference spectra of the participant in Fig. 3, which were obtained by subtracting the pre-^13^C spectrum from the post-^13^C spectra. Due to repositioning of the participant after oral administration of glucose, there were significant subtraction errors clearly visible at the NAA, tCr, and tCho singlet peaks.

Supplementary Figure S2 online displays the reprocessed time-course spectra of a participant in the previous study^11,12^, in which ^1^H spectra with TE = 106 ms were acquired from the prefrontal cortex of eight healthy participants after intravenous infusion of [U-^13^C_6_]glucose. In the fitted Gln time-course spectra, the Gln H4 peak is relatively weak. As a result, the gradual drop in peak amplitude of Gln H4 and the gradual rise in peak amplitude of Gln ^13^C satellites (vertically scaled up by a factor of 4) after intravenous infusion of [U-^13^C_6_]glucose are also very weak, which leads to relatively large errors in the computed ^13^C enrichment values of Gln C4. Supplementary Figure S3 online displays the plots of ^13^C enrichments of Glu and Gln vs. time after intravenous infusion of [U-^13^C]glucose for all eight healthy participants in the previous study. The ^13^C enrichment curves of Glu are relatively smooth and consistent with each other, indicating reliable measurement of ^13^C labeling of Glu. However, the ^13^C enrichment curves of Gln have large variations within each curve and between different participants. The end-point ^13^C enrichments of Glu and Gln for the eight participants computed from the last two post-^13^C spectra of each participant were found to be 51 ± 9% for Glu and 43 ± 16% for Gln. The last two post-^13^C spectra for the eight participants were acquired at 77 ± 9 min after intravenous infusion of [U-^13^C]glucose using the TE = 106 ms pulse sequence^11^. In comparison, a more reliable measurement of ^13^C-labeling of Gln was achieved using TE = 56 ms as evidenced by the higher Gln peaks in Fig. 3 and less scattered Gln ^13^C enrichment curves in Fig. 5.

## Discussion

Here, we demonstrated the feasibility of using a proton-only MRS technique (TE = 56 ms) to measure ^13^C enrichments of Glu and Gln in the dACC of healthy participants after oral administration of [U-^13^C]glucose. Compared to the existing indirect ^1^H-[^13^C] MRS techniques that use ^1^H and ^13^C surface coils, this ^1^H MRS method can acquire MRS data from a voxel away from the neocortex using a standard ^1^H head coil.

This method used both the pre-^13^C and post-^13^C MRS data to compute ^13^C enrichments of Glu and Gln. Because the participants exited the scanner after the pre-^13^C scan for oral administration of glucose and reentered the scanner for acquisition of the post-^13^C spectra, the pre-^13^C and post-^13^C spectra generally had small differences in metabolite linewidth and lineshape, as well as in the spectral baseline. In the previous work (TE = 106 ms)^12^, the participants stayed in the magnet during the entire scan. Frequency shift, zero-order phase, and line-broadening of the pre-^13^C spectrum were adjusted to fit each post-^13^C spectrum before generating a difference spectrum. The ^13^C-labeled Glu and Gln concentrations were obtained by fitting the difference spectrum. In the current study, the pre-^13^C and post-^13^C spectra generally do not match very well due to repositioning of the participants after oral glucose administration outside the magnet. As shown by Supplementary Fig. S1, subtraction of the pre-^13^C spectrum from the post-^13^C spectra caused significant subtraction errors. A novel post-processing method was developed in this work. The metabolite concentration ratios (/[tCr] + 3[tCho]) of acetate (Ace), NAA, N-acetylaspartylglutamate (NAAG), GABA, Glu, Gln, glutathione (GSH), Asp, total creatine (tCr), total choline (tCho), taurine (Tau), myo-inositol (mI), and scyllo-inositol (sI) obtained by fitting the pre-^13^C spectrum were used as constraints when fitting the post-^13^C spectra. Meanwhile, the spline baseline obtained from the pre-^13^C spectrum was also used in fitting the post-^13^C spectrum, along with an additional much weaker baseline. This approach of using the prior information from the pre-^13^C spectrum in the fitting of each post-^13^C spectrum avoids spectrum subtraction and hence the resultant subtraction errors.

The turnover of NAA and GSH in brain is known to be much slower than that of Glu and Gln. Therefore, ^13^C labeling of NAA and GSH, in addition to GABA, was omitted from our spectral model. In this study, ^13^C labeling of Glu and Gln was extracted using information from both the decrease in the pseudo singlet signals and the increase in their ^13^C satellite signals. The relationship between the parent Glu H4 signal and its ^13^C satellites is established by full density matrix simulations. In Fig. 1, the ^13^C satellite signals are much weaker than their parent signals for the same concentration. Therefore, the contribution from the ^13^C satellites to spectral fitting results is less than the corresponding parent signals. Our current approach simplifies the spectral fitting process by omitting the ^13^C labeling of GABA and adjusting the lineshape and linewidth to account for changes in B_0_ inhomogeneity and additional ^13^C labels. An alternative and more sophisticated approach is to use the actual ^1^H-^13^C couplings to compute the lineshape and linewidth of all signals since the outcome of the entire process of ^13^C labeling of Glu and Gln is determined by very few kinetic parameters such as the tricarboxylic acid cycle rate and the Glu-Gln cycle rate. This more sophisticated approach will be developed to improve the current technique. The high sensitivity of the proton MRS method employed in this study also suggests that it may be possible to use the current strategy to measure the initial rates of ^13^C incorporation into Glu and Gln during intravenous infusion of ^13^C-labeled substrates such as [1-^13^C]glucose, [2-^13^C]acetate, [3-^13^C]lactate, or [2,4-^13^C_2_]β-hydroxybutyrate.

The data acquired from the previous study^11,12^ were reprocessed using the new post-processing method. However, the ^13^C enrichment value of Gln was still highly scattered due to the small Gln resonance signals at TE = 106 ms. Using the current sequence (TE = 56 ms), the Gln H4 peak is at least 61% higher than that of the previous sequence (TE = 106 ms)^13^. Therefore, a more precise measurement of the end-point ^13^C enrichment of Gln was achieved in this study, which is evidenced by the more consistent ^13^C enrichment curves of Gln.

Although the current study used a 7 Tesla scanner to resolve Glu and Gln H4 signals in the ^1^H MRS spectra, spectral resolution of Glu and Gln H4 signals at 3 Tesla is also achievable^18^. In principle, it is possible to use a similar strategy to measure ^13^C labeling of Glu C4 and possibly Gln C4 using ^1^H MRS on the more prevalent 3 Tesla scanners. Research along this direction is currently in progress in our laboratory.

Previous studies have demonstrated quantification of the Glu-Gln neurotransmitter cycling flux between neurons and astroglia using direct ^13^C MRS by measuring ^13^C labeling of Glu and Gln at isotopic steady state following administration of ^13^C-labeled acetate or by measuring dynamic turnover of Glu and Gln following administration of ^13^C-labeled glucose, lactate, or β-hydroxybutyrate^1,19^. Therefore, it is possible to use proton-only MRS techniques to quantify the Glu-Gln neurotransmitter cycling flux with much higher spatial resolution and from brain regions inaccessible to surface coils, e.g., from limbic structures which play a major role in many neuropsychiatric disorders. As shown by Fig. 5, the transient isotopic steady state was approached but not attained in this study. Future studies should measure the initial rates, or the entire time course, or delay the time window of sampling the turnover curves after oral glucose administration to capture the transient isotopic steady state.

In summary, a proton-only MRS technique that induces intense Glu and Gln H4 singlets at TE = 56 ms was used to measure ^13^C enrichments of Glu and Gln in the dACC of five healthy participants after oral administration of [U-^13^C]glucose. A novel post-processing method was developed, in which the metabolite ratios and spline baseline obtained from fitting the pre-^13^C spectrum were used in the fitting of the post-^13^C spectra to compute the ^13^C enrichments of Glu C4 and Gln C4. At 113 ± 9 min after oral administration of [U-^13^C]glucose, the end-point ^13^C enrichment of Glu C4 was found to be 64 ± 5% (n = 5) and that of Gln C4 was found to be 40 ± 10% (n = 5). This technique offers a novel option to study Glu neurotransmission in the human brain with the high sensitivity and spatial resolution of ^1^H MRS using standard commercial equipment. ^13^C labeling in brain regions inaccessible to surface coils can also be investigated using proton MRS.

## Methods

Five healthy participants (two females, three males; age = 34 ± 12 years) were recruited for the study. Written informed consent was obtained from the participants before the study following the procedures approved by the Institutional Review Board (IRB) of the National Institute of Mental Health (NIMH; NCT00109174). The ^13^C enriched glucose solution was prepared by the National Institutes of Health (NIH) Clinical Center Pharmacy Department. All experimental protocols and methods were performed in accordance with the guidelines and regulations of NIH MRI Research Facility. Experiments were carried out on a Siemens Magnetom 7 Tesla scanner equipped with a 32-channel receiver head coil. The participants underwent overnight fasting before the MRS study.

In each scan session, the participant was first scanned to acquire the pre-^13^C MRS data. T_1_-weighted magnetization prepared rapid gradient echo (MPRAGE) images were acquired with repetition time (TR) = 3 s, TE = 3.9 ms, matrix = 256 × 256 × 256, and resolution = 1 × 1 × 1 mm^3^. Based on the MPRAGE images, the MRS voxel with a size of 3.5 × 1.8 × 2 cm^3^ was placed in the dACC of the participant. The first- and second-order B_0_ shimming coefficients were adjusted, achieving water linewidths of 11.1 ± 0.4 Hz. The pre-^13^C MRS scan was subsequently performed using a previously described pulse sequence^13^. The main component of the pulse sequence was a point resolved spectroscopy sequence (PRESS) with a 10 ms truncated 180° Gaussian pulse applied at 2.12 ppm. The pulse sequence parameters were: TR = 2.2 s, TE = 56 ms, spectral width = 4000 Hz, number of data points = 1024, number of averages = 264, number of unsuppressed water signal averages = 2, and total scan time = 10 min.

After the pre-^13^C MRS scan was finished, the participant exited the scanner and was orally administered 20% w/w 99% enriched [U-^13^C]glucose solution at a dosage of 0.75 g [U-^13^C]glucose per kg of body weight following procedures described in our previous study of carbonic anhydrase-catalyzed ^13^C magnetization transfer and references therein^17^. After a rest period, the participant reentered the scanner. The MPRAGE images were repeated, based on which the MRS voxel was placed at the same location and with the same size as in the pre-^13^C MRS scan. Post-^13^C MRS scans were repeatedly performed, each lasted 5 min (number of averages = 132). B_0_ shimming coefficients were adjusted before each MRS scan.

The pre-^13^C MRS data were processed first and the process was similar to that of the previous work^13^. Briefly, the raw free induction decay (FID) data were reconstructed into the pre-^13^C spectrum after going through the necessary steps that include multi-channel data combination^20^, eddy current correction^21^, Bloch-Siegert phase shift correction^22^, frequency drift correction^23^, and Fourier transform. The reconstructed pre-^13^C spectrum was fitted in the range of 1.8–3.4 ppm by linear combination of numerically computed basis spectra of Ace, NAA, NAAG, GABA, Glu, Gln, GSH, Asp, tCr, tCho, Tau, mI, and sI, as well as a spline baseline with 13 knots. Chemical shifts and coupling constants were obtained from the literature for GABA^24^, GSH^14^, and the rest of the metabolites^25^. The fitting program was developed and improved in-house and was based on the Levenberg–Marquardt least square minimization algorithm. After the metabolite concentrations in arbitrary unit were obtained from the fitting, we computed the metabolite ratios, which were defined as the concentration of a metabolite divided by the sum of concentration of tCr and three times the concentration of tCho. The combined concentration, [tCr] + 3[tCho], weighs approximately equally the intensities of the tCr and tCho singlet peaks and is less prone to errors than using either [tCr] or [tCho] alone.

The post-^13^C spectra acquired after oral administration of [U-^13^C]glucose were reconstructed in the same way as the pre-^13^C spectrum. For the subsequent fitting process, additional basis spectra of ^13^C-labeled Glu, Gln, and Asp were simulated. Additional ^13^C chemical shifts and ^1^H-^13^C and ^13^C-^13^C coupling constants were obtained from Ref.^16^ with the exception of long-range ^1^H-^13^C couplings involving the carboxylic carbons. As shown in Fig. 1, contribution from GABA is minimal. Hence, ^13^C-labeled GABA was not included in spectral fitting. When fitting each post-^13^C spectrum, the metabolites ratios (/[tCr] + 3[tCho]) of Ace, NAA, NAAG, GABA, GSH, Asp, tCr, tCho, Tau, mI, and sI were fixed to the pre-^13^C values. However, the linewidths and lineshape of the metabolites in a post-^13^C spectrum were allowed to be different from those of the pre-^13^C spectrum because participant repositioning, B_0_ shimming, and additional ^1^H-^13^C couplings caused changes in the linewidths and lineshape. The sum of metabolite ratios of Glu and ^13^C-labeled Glu was constrained to be the same as the metabolite ratio of Glu obtained from the pre-^13^C spectrum. The same constraints were also applied to Gln and Asp. Because the spectral baseline in the post-^13^C spectrum was expected to be slightly different from the spectral baseline in the pre-^13^C spectrum due to participant repositioning and B_0_ shimming, the spectral baseline in each of the post-^13^C spectrum was approximated by the sum of the spline baseline in the pre-^13^C spectrum and another much weaker spline baseline with 8 knots.

After the metabolite concentrations were obtained by fitting each post-^13^C spectrum, the ^13^C enrichment of Glu C4 for the post-^13^C spectrum was computed as the ratio of the concentration of its ^13^C satellites to the total concentration of Glu.

For comparison purposes, the MRS data from the previous study^11,12^ were reprocessed using the new post-processing method as described above. The MRS data were acquired using the pulse sequence^11^ with TE = 106 ms from the prefrontal cortex of eight healthy participants (5 females and 3 males, age = 37 ± 8 years). The pulse sequence used the following parameters: voxel size = 2 × 2 × 2 cm^3^, TR = 2.5 s, TE = 106 ms, J-suppression pulse frequency = 4.38 ppm, J-suppression pulse flip angle = 90°, spectral width = 4000 Hz, number of data points = 2048, number of averages = 128, and total scan time = 5.5 min for each individual spectrum.

## Supplementary Information

Supplementary Figures.

## Data Availability

Data are available upon request from the corresponding author Li An.
