# Supplementary material for: Detection of 13C labeling of glutamate and glutamine in human brain by proton magnetic resonance spectroscopy
Source: Sci Rep. 2022 May 24;12:8729. doi: 10.1038/s41598-022-12654-y (PMC9130156; doi:10.1038/s41598-022-12654-y)
Supplement: Supplementary file 1 — Supplementary Figures. [file 41598_2022_12654_MOESM1_ESM.docx]

**
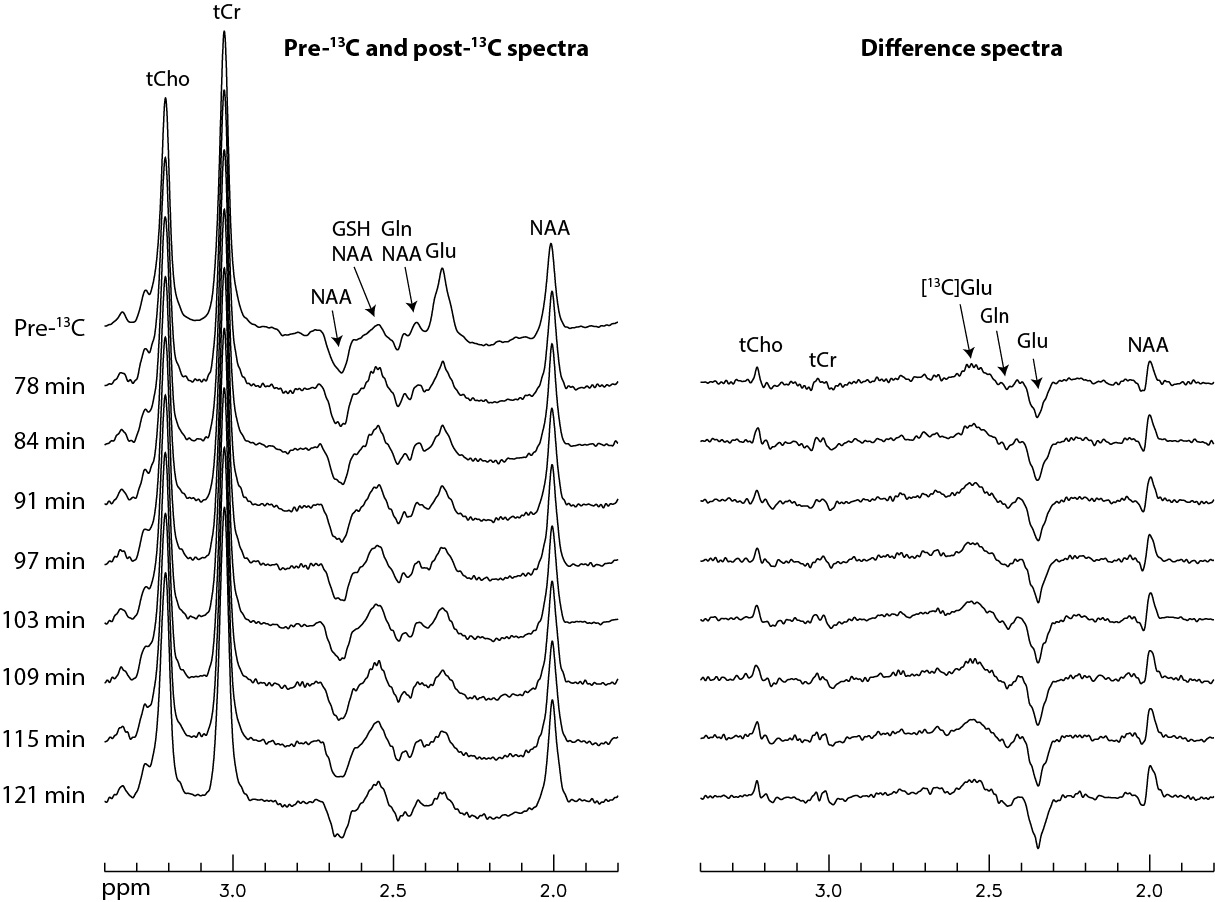
**

**Figure S1** Time-course spectra and difference spectra acquired from the dACC of the participant in Fig. 3. Note the significant subtraction errors visible at the NAA, tCr, and tCho singlet peaks.


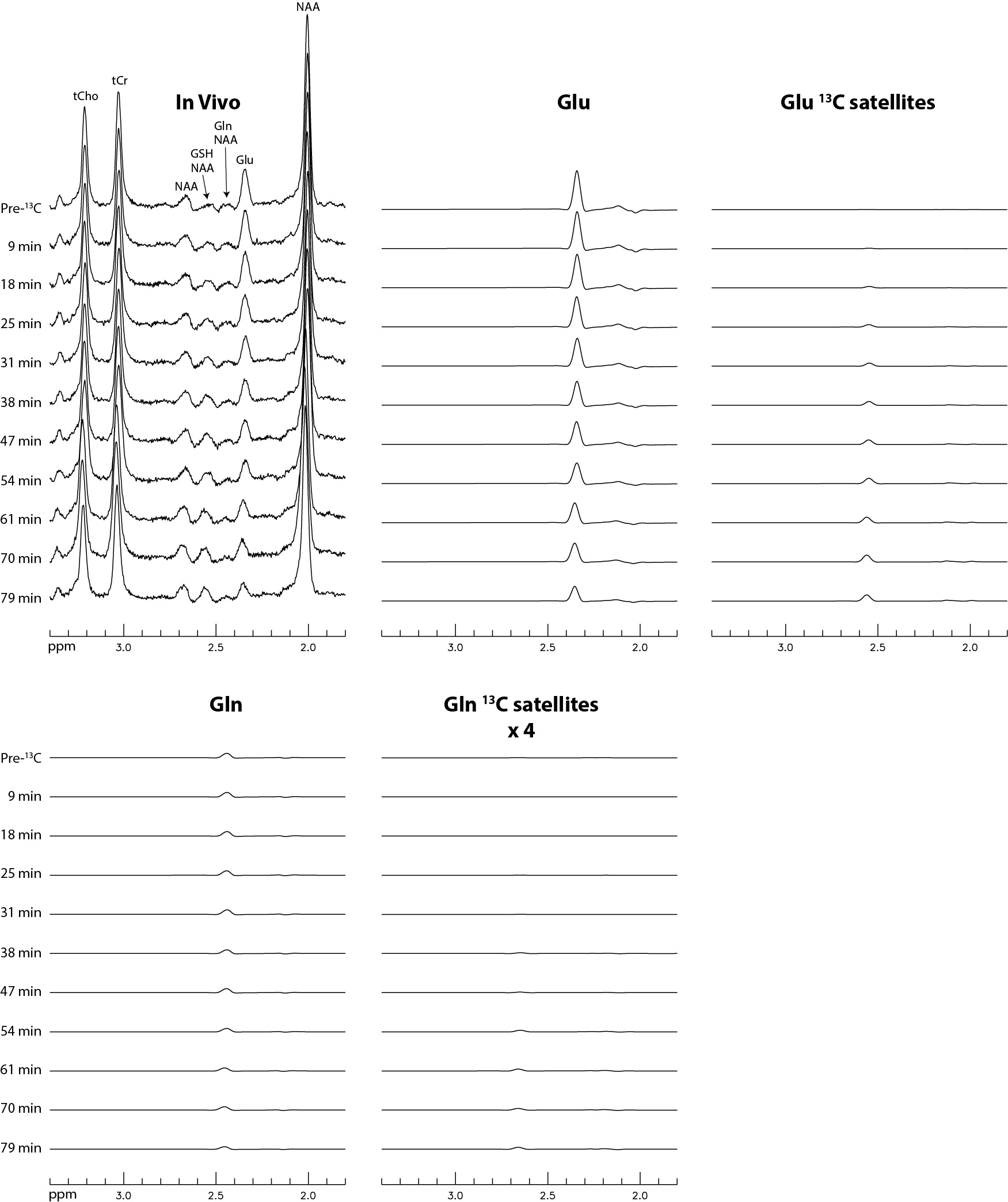


**Figure S2** Time-course spectra and corresponding fitted spectra of Glu, Gln, and their ^13^C satellites acquired from the prefrontal cortex of a representative participant in the previous study [^11^](#_ENREF_11)^,^[^12^](#_ENREF_12). The spectra for Gln ^13^C satellites have been scaled up vertically by a factor of 4. Voxel size = 2 × 2 × 2 cm^3^; TR = 2.5 s; TE = 106 ms; J-suppression pulse frequency = 4.38 ppm; J-suppression pulse flip angle = 90^°^; spectral width = 4000 Hz; number of data points = 2048; number of averages = 128 and total scan time = 5.5 min for each individual spectrum.


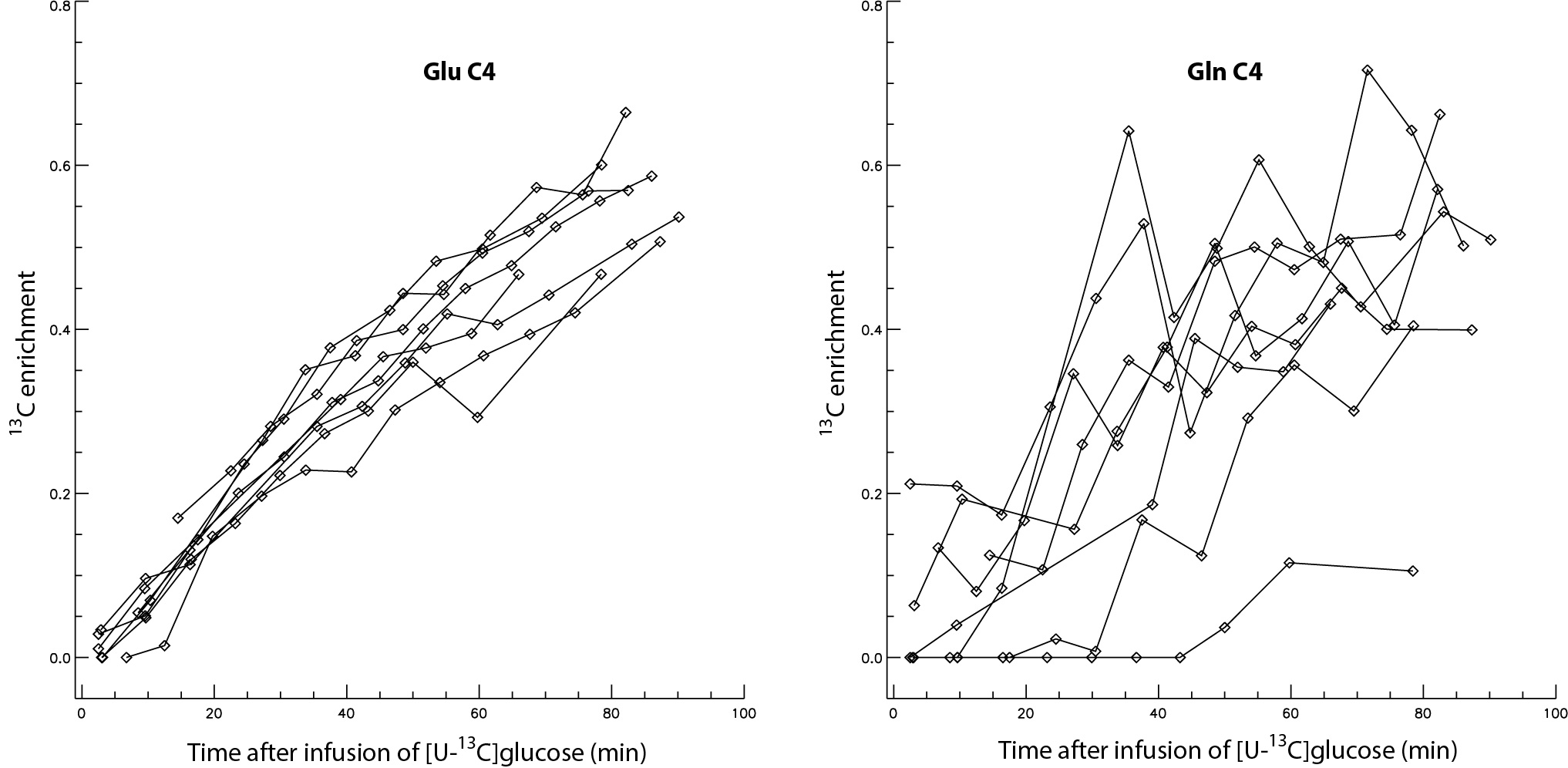


**Figure S3** Plots of ^13^C enrichments of Glu C4 and Gln C4 vs. time after intravenous infusion of [U-^13^C]glucose for eight healthy participants in the previous study.
